# Digital Finance: Reaching New Frontiers

**DOI:** 10.12688/openreseurope.15386.1

**Published:** 2023-02-23

**Authors:** Joerg Osterrieder, Branka Hadji Misheva, Marcos Machado

**Affiliations:** 1 Bern Business School, Bern, Switzerland; 2 Department of High-Tech Business and Entrepreneurship, University of Twente, Enschede, The Netherlands

**Keywords:** Machine Learning, Artificial Intelligence, Digital Finance, Financial Policies, Finance

## Abstract

Digital Finance must become the center of academic research in finance if the European financial industry is to remain competitive in the future. We argue that the new interdisciplinary field of Digital Finance should be prioritized based on the strategic priorities of the European Union, the needs of the finance industry, and the academic research gaps. Digital Finance as an interdisciplinary field will contribute to the strategic priorities of the European Union, such as financing for growth and jobs, financial stability and supervision, financial education, financing for small and medium-sized enterprises, and combating exclusion and inequality in access to credit.

## Introduction and timeliness of Digital Finance for Europe

Digital Finance must become the center of academic research in finance if the European financial industry is to remain competitive in the future. We argue that the new interdisciplinary field of Digital Finance should be prioritized based on the strategic priorities of the European Union, the needs of the finance industry, and the academic research gaps. Digital Finance as an interdisciplinary field will contribute to the strategic priorities of the European Union, such as financing for growth and jobs, financial stability and supervision, financial education, financing for small and medium-sized enterprises, and combating exclusion and inequality in access to credit.

In addition, we propose two tasks for the interdisciplinary field of Digital Finance: (1) Analyze which markets should be affected by the application of Digital Finance and what risks are associated with its failure to reach certain segments of the market, particularly in terms of financial inclusion, youth finance, and sustainable development. (2) Identify and investigate the potential for positive externalities between Digital Finance and other research fields, including Machine Learning and Artificial Intelligence, Sociology of finance, Globalization, Monetary policy, Macroeconomics; Social network analysis; Quantitative Economics, Banking and Finance; Policy; Law and Economics.

The European Union should aim for a unified approach to Digital Finance, such as the establishment of a European Digital Finance Research Lab that fosters interdisciplinary collaboration among researchers from various fields.

A competitive European financial sector is vital for the modernisation of the European economy across sectors and to turn Europe into a global digital player. The term Digital Finance refers to the rapid development of new technology, goods, and business models that have taken place in recent years.

We have identified the five most pertinent areas within this domain:

- *Towards a European financial data space.*
- *Artificial intelligence for financial markets.*
- *Towards explainable and fair decisions generated by Artificial Intelligence (AI).*
- *Driving digital innovations with Blockchain applications.*
- *Sustainability of Digital Finance.*

What they have in common:

- They are all key strategic priorities of the European Commission over the next five years ^1^.
- They contribute to the UN Sustainable Development Goals ^2^.
- Europe must invest significantly in them over the next five years if it is to remain globally competitive.
- They are characterised by a significant shortage of skilled labour.
- Initial progress has been made in academia, but there are still numerous unanswered research questions.
- They have the potential to revolutionise the Finance industry with new technologies, business models, and products, while strengthening the resilience of Europe.
- They are the foundation for a new generation of PhD candidates and training in Digital Finance.

Considering these developments across industries and within the financial sector, it is absolutely essential to work on those research topics now because:

- Digital Finance has already changed the way the Finance industry works.
- To deal with the realities of academia and industry, researchers in Finance will be required to acquire the skill set of Digital Finance.
- There is a substantial research gap in academia that needs to be resolved now by academics and a new generation of Digital Finance PhDs to keep Europe's Finance industry competitive.

Today, Digital Finance does not exist as a standalone research discipline, despite many research gaps, the EU’s key strategic priorities and the urgent needs from industry.

To overcome this and significantly advance the methodologies and business models for Digital Finance, five main objectives are targeted:

- **Towards a European financial data space.** Ensure sufficient data quality to contribute to the EU's efforts of building a single digital market for data.
- **Artificial intelligence for financial markets.** Address deployment issues of complex artificial intelligence models for real-world financial problems.
- **Towards explainable and fair AI-generated decisions.** Validate the utility of state-of-the-art explainable artificial intelligence (XAI) algorithms to financial applications and extend existing frameworks.
- **Driving digital innovations with Blockchain applications.** Design risk management tools concerning the applications of the Blockchain technology in Finance.
- **Sustainability of Digital Finance.** Simulate financial markets and evaluate products with a sustainability component.

In the rapidly evolving field of Digital Finance, multiple disciplines are required that go substantially beyond the traditional Finance research in a wide range of inter-sectoral applications: data quality, AI and Machine Learning (ML), Explainability of AI (XAI), Blockchain applications and sustainable finance; all of which are required for a wide range of industrial (financial products, risk management, customer-centric products, enhanced processes, and improved services) and scientific (new AI techniques, new business models, and enhanced modelling) applications, necessitating new scientific insight, new training courses, and future specialists in the field.

Academia and Industry need to work together. The European Finance industry needs to compete on a global scale. To overcome key hurdles which financial service companies will face in the near future, they will have to find answers to the below (WEF 2020 ^3^):

- Data quality issues related with the increasing dimensionality of financial data.
- Deployment issues of complex models in real-world applications.
- Deficits in trust and user adoption of AI-supported financial products.
- Potential data or algorithmic bias inherent in AI models.
- Labour shortage: AI leaders overwhelmingly argue that access to talent represents a key obstacle to the digitization efforts in finance, as more sophisticated solutions demand different employee capabilities.

To achieve scientific, societal and economic/ technological impact, all of those hurdles have to be solved.

The strategic priorities of the European Union, industrial needs and academic research gaps lead to the scope of a required research programme for Digital Finance:

- One has to focus on proposing novel methodologies and applications to address the key data quality issues associated with high-dimensional, high-variety, and high-velocity datasets. In addition, deployment issues with cutting-edge machine learning, deep learning, and reinforcement learning methods must be addressed in order for methodological advances to be useful in real-world applications.
- The most significant obstacles to industry adoption of technological innovation have to be addressed.
- The principles of a trustworthy and secure AI for financial applications have to be established and the methodologies have to be defined that can satisfy the explainability requirements of various stakeholders within the financial sector. The outputs will be central to the applicable regulation of this technology.
- The efficacy and impact of Blockchain adoption in financial markets need to be examined and risk management tools need to be proposed to address some of the main concerns around this technology (e.g., financial stability and fraud detection). Additionally, a Blockchain industry standard needs to be implemented, and various perspectives have to be explored.
- We need to focus on sustainable financial products, on simulating markets and designing recommender systems to analyse the effects of adopting principles of sustainable finance philosophy.

All research topics must be handled collectively for an integrated view of Digital Finance.

The research agenda for Digital Finance can be grouped into five inter-connected topics.

Data is in the centre of the digital transformation (A European strategy for data, European Commission 2020 ^4^), though utilising it is accompanied by many challenges. By ensuring that all dimensions (accuracy, consistency, completeness, currency, volatility and timeliness) of data quality are satisfied, that we have sufficiently large, high-quality data available and that we can detect dependencies in high dimensions, high frequency and high veracity financial data, we are in a position to move towards a European financial data space and make use of substantially more data sources than today.

AI models need to be deployed. AI has improved decision making in numerous areas, including risk management, compliance (anti-money laundering, fraud prevention, KYC), trading strategies and personalised banking and advice (A European approach to AI, European Commission 2021 ^5^), and yet, adoption of AI-based tools in practice has still been rather slow.

With the required research agenda, we will describe the primary challenges and opportunities for industry's adoption of technological development, encourage a larger deployment of state-of-the-art ML models in real-world financial applications and simulate the market environment in Reinforcement Learning (RL) applications for market applications, thereby removing the primary barrier to the application of this technology in finance.

Deployed AI models need to be explainable, trustworthy and unbiased. AI-driven innovation can bring enormous benefits but such complex solutions are often referred to as “black boxes” because typically it is difficult to trace the steps the algorithms took to arrive at its conclusions (The European Approach to Excellence and Trust, European Commission 2020 ^6^).

We will describe how well XAI tools meet the explainability requirements of various financial value chain stakeholders, develop non-perturbation-based XAI methods that preserve the natural time ordering and dependence structures of the data and create methodologies to ensure that algorithmic systems do not produce socially biased outcomes that exacerbate inequalities. The Blockchain technology is needed for digital innovations. Blockchain is another driver of the technological change of the financial ecosystem (The European Commission’s Blockchain strategy, 2021 ^7^), though there are no systematic studies that assess whether the benefits outweigh the costs. We need to analyse the efficiency of financial service providers that adopt the Blockchain technology, contribute to more robust and efficient financial markets by understanding how to tokenize financial assets, reduce the risk of fraud and highly volatile crypto assets and establish a global, industrial standard for the architecture of Blockchain-based financial systems.

Digital Finance needs to become more sustainable. Climate change and environmental degradation are becoming an existential threat to Europe and the world at large (European Green Deal, 2019 ^8^), though inevitably, transitioning to a sustainable future with inclusive, green economies and resilient ecosystems is associated with many challenges.

We will make financial strategies for sustainable investing more objective, optimised, integrated, and operational, measure the social and environmental effects of sustainable finance, define the primary characteristics of sustainable financial assets and instruments and overcome the most significant obstacles to implementing sustainable finance strategies.

Such a research framework is tightly integrated into the European Digital Finance Package (EC 2020 ^9^), and will contribute to one of the four EU’s key strategic orientations ^10^, namely the promotion of an open strategic autonomy through the development of key digital, enabling and emerging technologies, sectors and value chains that further accelerate the digital and green transition of Europe.

## State-of-the-art research in digital finance and beyond

Both academia and industry are increasingly faced with data characterised with a staggeringly high number of dimensions, high variability and high veracity. Utilising such data is accompanied by many challenges.

We need to apply an unprecedented collection of innovative data quality methodologies and data augmentation techniques to a wide variety of industry-relevant datasets. Overcoming the obstacles of data quality and availability (through novel or extended NLP methods ^11^, deep generation of data ^12^, and anomaly- and dependence detection models baked on network concepts) will contribute to the literature and further provide a valuable tool for the European Finance industry to enhance product offerings, reduce financial market risks, and work toward a European financial data space.

The deployment of complex AI models to pertinent financial problems is needed. For financial applications such as risk management, trading strategies, and client-centric financial products, AI models trained and tested in closed, academic settings have shown great promise. Yet, real-world applications (in open environments) are more challenging. Using industry-ready use cases, the viability of novel dynamic rating models ^13^, automated trading platforms ^14^, and market environments for RL agents ^15^ in real-industry settings needs to be demonstrated for the first time.

A first-of-its-kind qualitative analysis of the primary obstacles to deploying innovative technologies in industry is needed and new solutions for resolving these obstacles need to be proposed which in turn is a crucial step towards a widespread adoption of complex models in the financial sector.

The crucial question of how to build trust in human-centric AI models needs to be addressed as opposed to the currently widespread AI black boxes ^16^, which do not meet the modern European requirements of explainability, trust and unbiasedness. The precise methodologies for explainable AI will depend heavily on the specific prototype models by industry that are in scope.

The applicability of state-of-the-art XAI algorithms to financial applications needs to be validated and XAI frameworks need to be extended, ensuring that complex models applied to financial use cases satisfy the explainability requirements of different stakeholders within the finance value chain and do not reinforce social biases. A qualitative evaluation of the comprehensive frameworks' insights into explainability needs to be made in comparison to the baseline models. Through industry-ready use cases, the viability of the proposed framework for audience-dependent explanations ^17^, the novel time-series XAI methods, and the fair algorithmic designs ^18^ should be explained.

Based on the correct use and management of data, as well as the expansion of various trustworthy and explicable AI tools, Blockchain technologies can then be used to advance the frontiers of financial digital innovation. New Blockchain-based tools are essential inputs for a new IT-infrastructure that can be implemented in a variety of IT domains. These infrastructures should account for the integration of multiple data sources, define a standard dictionary, eliminate ambiguity, and permit other teams to access all customer data from a centralized repository to ensure interoperability. The quality of the proposed Blockchain tools ^19^ must be ensured by defining and monitoring efficiency measures. Additionally, a qualitative evaluation should be conducted by comparing various frameworks. Concurrently, research related to this topic should focus on proposing novel risk management solutions to some of the primary concerns surrounding Blockchain applications in finance, such as fraud detection and financial stability. Various strategies can be employed for this purpose, such as utilizing network theory concepts and a set of exploratory tools to improve statistical models to develop industry-ready use cases for fraud detection in financial networks ^20^ and to propose a comprehensive and dynamic risk index for cryptocurrencies ^21^.

Finally, sustainable finance topics should be scrutinized from various angles. For example, studies should simulate and evaluate markets to replicate the relationship between banks, financial institutions, and their retail and business clients in a sustainable environment that takes into account various factors (e.g., Green AI ^22^ and green credit score ^23^). Numerous indicators and metrics, including loan interest rate, customer attrition, CO2 emissions, customers' access to credit, and firms' profits, must be compared in order to compare, analyze, and evaluate these market simulations ^24^. The analysis of long-term financial growth will inform the development and modification of industry policies and strategies. This will ensure that sustainability is an integral component of the Digital Finance industry, thereby contributing to the European Green Deal.

## Overall methodology for Digital Finance

The central research question for Digital Finance is how innovative technologies, like Big Data, AI and Blockchain, can be used to support Digital Finance in view of the emerging complexities: (i) high-dimensional, high-variety, high-velocity dataset; (ii) limited samples of high-quality data to train various ML models, (iii) no comprehensive pipeline for building and deploying complex ML models in real settings, (iv) no explainability techniques that are specifically tailored to financial datasets and satisfy the explainability needs of various financial stakeholders, (v) no industry standard for Blockchain applications and (vi) no common ESG scoring framework.

All of these complexities are methodological in nature and in order to tackle them, a pragmatic, data-focused, inductive research using a combination of research strategies (case studies, experiments and actions) and methods enriched with continuous cooperation with a feedback loop from industry and regulators needs to be carried out. We elaborate on the central layer of the research methodology, which combines academia, industry, dissemination, and training by structuring it into data and methodology as well as the five topical themes.

1.   Data. All research objectives heavily depend on the access to financial data (both structured and unstructured) for the different functions of finance: trading (including new assets like cryptos), personal lending, SME lending (small and medium-sized enterprises) and sustainable investing. Industry and academia possess a plethora of datasets that have not yet been fully exploited for academic research and business use. Additionally, researchers will need to carry out primary data collection as well i.e. collect and generate new relevant datasets (e.g. through web scraping, surveys and questionnaires).

2.   Two-phased methodological approach. Digital Finance needs to emphasise a mixed-method approach, resulting in comprehensive research findings that incorporate qualitative and quantitative data signals. Typically, each research project consists of two stages. In the initial phase, existing methods need to be applied to new datasets and transform them into prototypes and use cases so that new financial products can be developed. Once those MVPs are available, additional research is needed to achieve new breakthroughs and methods in a second phase.

**Towards a European financial data space.** In order to address the growing complexity of financial data, researchers need to use natural language processing (NLP), transformers and attention networks to incorporate text and identify temporal dependencies in various financial use cases. On the topic of data augmentation, researchers need to look at convolutional networks with attention and transformers for simulating various financial time series.

**AI for financial markets.** In order to address some of the key deployment issues of AI models in real financial use cases, researchers need to work on: simulating the real market environment in deep reinforcement learning applications; produce dynamic scoring systems based on complex ML models (XGBoost, random forest, SVM, neural nets etc.) that are able to deal with sparse credit history of loan applicants; and apply (unrestricted-) mixed data sampling ((u-) midas) methods so to align different frequency financial data.

**Towards explainable and fair AI-generated decisions.** In order to validate the utility of classical XAI methods for finance, researchers need to map the state-of-art post-hoc global and local explainability techniques (LIME, SHAP, LRP) to the explainability needs of finance stakeholders. IRPs will also focus on developing new XAI methods for time series that mark preference for the sensitivities or partial derivatives for each explanatory variable included in the ML’s specification, thus excluding the need for perturbation. Finally, to tackle the challenges surrounding algorithmic bias, disparate learning processes (DLPs) and the variational fair Autoencoder, will be applied.

**Driving digital innovation with Blockchain applications.** Researchers need to propose a novel methodology for an industry-level Blockchain standard which includes comparison criteria, documentation criteria and content criteria. Furthermore, in order to tackle the emerging risks from Blockchain applications in finance (volatility of crypto assets and fraud), researchers will need to develop a dynamic crypto risk index based on a model selection criteria, and propose network-community detection models so to explore the transactional crypto network and uncover ambiguous links.

**Sustainability of digital finance.** Within a Digital Finance research agenda, researchers need to explore, train and compare content-based, collaborative and hybrid filtering approaches in the attempt to find the most suited recommender system to support financial institutions and customers on investing in sustainable technologies. Yet another methodological challenge that needs to be tackled is that of building unifying and comprehensive green credit scores for retail and business clients.

For a successful research program on Digital Finance, continuous cooperation and feedback loops are needed. Continuous collaboration and feedback loops with industry and other relevant non-academic partners are a defining characteristic of the required research model. Each research project needs to be established jointly by academic and industry representatives. The progress with respect to the established research objectives needs to be continuously monitored and validated by industry, and in accordance with the existing and upcoming technological regulations. The continuous feedback loop with industry ensures that developed solutions are applicable in the real world.

**Research challenges.** There are both data availability and methodological challenges. Data has already been used by academia and industry, but might not be sufficient. Then, data anonymization techniques need to be employed that enable data sharing or resort to synthetic data generation techniques (GANs). On the methodology-related challenges, in cases in which the planned techniques do not lead to the envisioned outcomes, the scope of models to be trained and tested needs to be expanded.

## An interdisciplinary approach to Digital Finance

Digital Finance necessitates the integration of methods, information, data, techniques, tools, perspectives, concepts, and theories from the seven essential disciplines. Those are Economics, Finance, Management, Economic Development, Computer Science and Informatics, Applied Mathematics, and Political Science.

Interdisciplinarity of Research Methods:

- Each research topic is developed at the intersection of multiple disciplines and requires input and feedback from academic experts conducting research in various fields.
- All research topics in Digital Finance have an applied focus, each addressing a specific obstacle companies face when deploying innovative financial technologies.
- Regulatory and supervisory bodies play a crucial role in defining the business models of financial service providers. To account for the regulatory/supervisory discipline as well, researchers need to validate their research through discussions with industry and regulators.

## Artificial Intelligence in Digital Finance

Digital Finance requires developing and employing a wide range of AI systems for use in Digital Finance, while adhering to the European Commission's AI ^25^ guidelines on a consistent basis. By design, technical robustness will be ensured. These systems have to be developed to become:

1.   Technically robust, accurate, reproducible, and able to handle and report on potential failures, inaccuracies, and errors in proportion to the risk posed by the AI-based system or technique. To ensure reproducibility and technical robustness, researchers need to develop AI models and rigorously test, validate, and apply them in various scenarios and conditions. They also need to address error recommendations or suggest alternative parameters for anticipated system failures. AI systems’ technical robustness needs to be monitored.

2.   Socially robust; they duly consider the context and environment in which they operate. Digital models detect systematic data biases and error-affected client groups. To ensure fairness, artificial intelligence will have to be monitored and optimised, and so that stakeholders can challenge the model's decisions. AI tools need to promote green AI. AI algorithms need to be trained in subsets of variables that predict a dependent variable to reduce training time and energy consumption (i.e., carbon footprint). AI tools have to be developed that are Human-Centric AI, having the Finance industry and end users in mind.

3.   Reliable and function as intended, minimising unintentional and unexpected harm, preventing unacceptable harm, and safeguarding humans' physical and mental integrity. The AI tools that are developed will need to undergo rigorous customer- and industry testing to minimise adverse effects.

4. Able to provide a suitable explanation of its decision-making process whenever an AI-based system can have a significant impact on people’s lives. This has to be accomplished by enabling systems that are explicable, trustworthy, and ethical. Legal accountability, transparency, and fairness need to be considered from the start of a project. Each research project needs to begin with ethical considerations. Each AI pipeline implementation needs to be exhaustively described upon completion.

## Impact

A new paradigm for Digital Finance will have profound effects on science, society, and technology. Digital Finance research will contribute significantly to all nine of the primary scientific, economic/technological and societal Key-Impact Pathways (KIPs) as defined by the European Union.

## Impact on policy and civil society

The entire Digital Finance agenda is driven by the EU's Digital Finance strategy. All outcomes will contribute immediately to this. The findings must be disseminated via policy papers, participation in policy hearings, and direct workshops with policymakers.

The core of the Digital Finance agenda is the protection and reduction of risk for end users. In addition to educating citizens about finance, it is necessary to reduce the risks of financial markets. New Digital Finance products are typically 30% less expensive than conventional products, which could have a consumer impact of millions of euros.

## Expected scientific impact

Digital Finance will generate significant scientific impact by: (i) producing research that contributes to Europe's long-term competitiveness, (ii) driving the digitalization of the financial industry and facilitating collaboration between universities, research centres, and industry, (iii) strengthening Europe's human capital in research and innovation by training doctoral candidates in multiple disciplines, using a novel interdisciplinary approach to Digital Finance, and by creating new, high-quality training modules on the intersection of digital technologies and finance (iv) embracing open science practices, innovation, entrepreneurship, gender equality, and transparency practices thus enabling the diffusion of the new knowledge generated.

## Expected societal impact

Research in Digital Finance will address EU policy priorities and global challenges. Digital Finance research will address the primary concerns of the EU Digital Finance policy by utilising data and AI to develop sustainable digital assets, products, and services with a positive social impact.

Digital Finance needs to be concerned with the delivery of benefits and impact through R&I missions. Digital Finance research will ensure that the development and deployment of AI models will take into account the European Commission's (EC) priority: the European Green Deal, a Europe fit for the digital age and the EU's mission of climate change adaptation. Specifically, the research will need to respect people's rights and promote mechanisms by which financial technology tools work to earn people's trust, with a particular focus on reducing biases based on gender and race. Referring to climate change, Digital Finance needs to contribute by adopting modelling, conducting additional research on sustainable finance, and disseminating its findings to the European community and the rest of the world. Digital Finance needs to enhance the social adoption of R&I. Digital Finance research needs to be committed to disseminating the results of each research project and any research that may intersect with these projects through the media of beneficiaries, industrial partners, and associated partners.

Digital Finance research needs to invest heavily in creating societal awareness through a participatory approach at the EU level, highlighting the added value of the new solutions for a variety of application domains and preparing society for action in response to digital finance transitions. In addition to the aforementioned, it is evident that Digital Finance research needs to significantly contribute to bringing new green, digital solutions to the market that can increase Europe's resilience and readiness for the digital age and the EU's mission to adapt to climate change.

In addition, open science needs to be adopted, and framework methods and examples need to be widely shared. By adopting open science practices, Digital Finance researchers will make data exploitable for industry participants through the development of advanced statistical methodologies; it will also support the financial system by offering an alternative to conventional financial intermediation. As a result, it will increase the use of more accurate estimates of creditworthiness, reduce the possibility of fraud, and strengthen consumer and investor protection.

## Expected economic and technological impact

The other three KIPs are designed to summarise the anticipated economic effects of the move towards Digital Finance. KIP7 refers to innovation-based growth generation. This contribution can be realised as a result of the interdisciplinary approach taken by various research projects to specific digital finance issues. Consequently, financial institutions and end users will have access to new products and services based on AI tools, in particular: New automated trading platforms, that enable wider access to the financial markets; systems for anomaly and fraud detection, that will help finance service providers operate more efficiently and avoid significant costs related with fraudulent behaviours in financial networks; predictive models for text, which in turn, enable more accurate models for forecasting financial trends (and new products based on that); XAI frameworks, that will enable end users to check the logic behind a ML-based decision that affected them; new crypto risk indices, which will help the general public and regulators understand how risk changes over time and support decision making; industry standard for Blockchain, which will enable trust in the technology; recommender systems for new sustainable investment products.

Sustainable finance will contribute to an intersectoral and interdisciplinary research agenda by enhancing the industry's efficiency. Through the intersection of data analysis, AI, computational sciences, financial engineering, and ethical regulations, Digital Finance research will enable the creation of more and better jobs. Lastly, research and innovation investments can be leveraged. The issuance of patents across various research projects comprising Digital Finance will encourage additional research in digital finance. Digital Finance will commit to providing models that reduce costs, improve efficiency, and increase profits, allowing financial institutions and end users to increase their investment in R&I.

## Conclusions for a new Digital Finance era

If Europe's financial sector is to stay competitive in the future, digital finance must become the focus of academic research in Finance. We believe that strategic aims of the European Union, demands of the financial sector, and academic research shortages should all inform the emerging multidisciplinary area of Digital Finance. Financing for growth and jobs, financial stability and supervision, financial education, financing for small and medium-sized enterprises, and addressing exclusion and inequality in access to credit are all strategic priorities of the European Union that will benefit from the multidisciplinary study of digital finance.

In addition, we two initiatives for the emerging subject of Digital Finance should be formed, which bridge several disciplines. (1) Assess the potential markets that will be impacted by the introduction of Digital Finance, and the risks associated with not reaching specific sectors of the market, especially in regards to financial inclusion, youth finance, and sustainable development. (2) Recognize and explore the synergies that may exist between Digital Finance and other areas of study, such as the sociology of finance, globalization, monetary policy, macroeconomics, the analysis of social networks, quantitative economics, banking and finance, policy, and law and economics.

The European Union should create a European Digital Finance Research Lab to promote multidisciplinary cooperation among experts from different sectors and work toward a uniform approach to Digital Finance. We've narrowed this field down to its top five areas of interest, which are:

- The move toward a unified European financial data market.
- The application of AI to the stock market.
- An effort toward AI-generated models that can be explained and are equitable.
- Pushing forward cutting-edge digital developments with useful Blockchain implementations.
- The Long-Term Viability of Digital Banking.

What they all have in common is that they are high-level strategic goals for the European Commission for the next five years and are aligned with the UN's Sustainable Development Agenda. If Europe wants to keep up with the rest of the world over the next five years, it must make considerable investments in these areas.

## Ethics and consent

Ethical approval and consent were not required.

## Data Availability

No data are associated with this article.

