# Supplementary material for: Digital Finance: Reaching New Frontiers
Source: Open Res Eur. 2023 Feb 23;3:38. [Version 1] doi: 10.12688/openreseurope.15386.1 (PMC10445873; doi:10.12688/openreseurope.15386.1)
Supplement: Peer review history

# Peer review: Reviewer response for version 1

**DOI:** 10.21956/openreseurope.16632.r30930

**Published:** 2023-03-20

**Authors:** Codruta Mare

**Affiliations:** 1 Department of Statistics-Forecasts-Mathematics, Faculty of Economics and Business Administration, Babeș-Bolyai University, Cluj-Napoca, Romania

The article addresses a very fashionable topic, that is currently given much attention even in the European Union's strategies - Digital Finance. It presents the field of Digital Finance in a reader friendly manner so that policy makers and stakeholders can also understand the issues related to it. The paper stresses the importance of Digital Finance for insuring the competitiveness of the European financial sector at international level. It points out the specificities of the field in respect to the data and methodologies used, along with defining and explaining five main objectives to be treated by research in this field.

 The paper presents very nicely the different types of impact that may appear. 

 Although I find the article very relevant for the field, and I enjoyed reading it, I do have some comments for the authors:

1. Rephrase either the abstract, or the first paragraph in the Introduction - now they are the same.
2. You identify the 5 most pertinent areas/ objectives. But you repeat them several times, which hinders the reading process. I suggest you to merge the 1st and 2nd time you present them, in order to increase the coherence of the article.
3. You accurately present that research in Digital Finance needs high-dimensionality data. Considering the field and its sensitiveness, I would insist more on XAI, on one hand, and on GDPR/data protection issues on the other. 

Is the argument persuasive and supported by appropriate evidence?

Yes

Is the work clearly and cogently presented?

Yes

Is the topic of the essay discussed accurately in the context of the current literature?

Yes

Does the essay contribute to the cultural, historical, social understanding of the field?

Yes

Reviewer Expertise:

Statistics and Econometrics, Economic Forecasting

I confirm that I have read this submission and believe that I have an appropriate level of expertise to confirm that it is of an acceptable scientific standard.

# Peer review: Reviewer response for version 1

**DOI:** 10.21956/openreseurope.16632.r30934

**Published:** 2023-03-14

**Authors:** Ioana-Florina Coita

**Affiliations:** 1 Department of Finance and Accounting, Faculty of Economics, University of Oradea, Oradea, Romania

The paper addresses many topics such as: interdisciplinary study and research of digital finance, policy priorities in the field, importance of explainability, accountability and auditability in ML models that are implemented in the sector of digital finance, hurdles and opportunities that industry needs to face regarding regulatory and technical aspects and using AI in fintech products and services, sustainable finance and others.

 The strong points refer to a thorough presentation of the topics addressed and the complexity of the digital finance sector pesented. A good blend of policy and research aspects make it usefull to either decision makers and specialists. The structure is coherent and covers many aspects of the subject. Conclusions are relevant to real context and current trends in digital and sustainable finance.

 Weak points refer to the fact that the abstract is the same as the 1st paragraph and that should be changed. The word 'must' in the abstract - I think it is too strong and needs revision. In my opinion the enumeration paragraphs and bullet points would look better if transformed in plain text. The diversity of sentences could be condensed in complex paragraphs that could follow several related ideas.

 Overall, the paper is very relevant, coherent and condensed. It covers some of the most important current trends in digital finance.

Is the argument persuasive and supported by appropriate evidence?

Yes

Is the work clearly and cogently presented?

Yes

Is the topic of the essay discussed accurately in the context of the current literature?

Yes

Does the essay contribute to the cultural, historical, social understanding of the field?

Yes

Reviewer Expertise:

finance, sustainable finance, fintech

I confirm that I have read this submission and believe that I have an appropriate level of expertise to confirm that it is of an acceptable scientific standard.
